# Detection of Marburg Virus Antibodies 25 Years After Outbreak in Watsa, Democratic Republic of the Congo

**DOI:** 10.1093/infdis/jiag155

**Published:** 2026-03-16

**Authors:** Sydney Merritt, Patrick K Mukadi, Jean Paul Kompany, Megan Halbrook, Kamy Musene, Skylar A Martin, Michael Beya, Merly Tambu, Teri Ann S Wong, Jean Jacques Muyembe-Tamfum, Didine K Kaba, Axel T Lehrer, Jason Kindrachuk, Nicole A Hoff, Placide Mbala-Kingebeni, Anne W Rimoin

**Affiliations:** Department of Epidemiology, Fielding School of Public Health, University of California, Los Angeles, California, USA; Department of Epidemiology and Global Health, Institut National de Recherche Biomédicale (INRB), Kinshasa, Democratic Republic of the Congo; Department of Medical Biology, University Hospital of Kinshasa, University of Kinshasa, Kinshasa, Democratic Republic of the Congo; Department of Epidemiology and Global Health, Institut National de Recherche Biomédicale (INRB), Kinshasa, Democratic Republic of the Congo; Department of Medical Biology, University Hospital of Kinshasa, University of Kinshasa, Kinshasa, Democratic Republic of the Congo; Department of Epidemiology, Fielding School of Public Health, University of California, Los Angeles, California, USA; Department of Epidemiology, Fielding School of Public Health, University of California, Los Angeles, California, USA; Department of Epidemiology, Fielding School of Public Health, University of California, Los Angeles, California, USA; Kinshasa School of Public Health (KSPH), University of Kinshasa, Kinshasa, Democratic Republic of the Congo; Department of Epidemiology and Global Health, Institut National de Recherche Biomédicale (INRB), Kinshasa, Democratic Republic of the Congo; Department of Tropical Medicine, Medical Microbiology and Pharmacology, John A. Burns School of Medicine, University of Hawaii at Manoa, Honolulu, Hawaii, USA; Department of Epidemiology and Global Health, Institut National de Recherche Biomédicale (INRB), Kinshasa, Democratic Republic of the Congo; Department of Medical Biology, University Hospital of Kinshasa, University of Kinshasa, Kinshasa, Democratic Republic of the Congo; Kinshasa School of Public Health (KSPH), University of Kinshasa, Kinshasa, Democratic Republic of the Congo; Department of Tropical Medicine, Medical Microbiology and Pharmacology, John A. Burns School of Medicine, University of Hawaii at Manoa, Honolulu, Hawaii, USA; Department of Medical Microbiology and Infectious Diseases, Max Rady College of Medicine, University of Manitoba, Winnipeg, Manitoba, Canada; Department of Epidemiology, Fielding School of Public Health, University of California, Los Angeles, California, USA; Department of Epidemiology and Global Health, Institut National de Recherche Biomédicale (INRB), Kinshasa, Democratic Republic of the Congo; Department of Medical Biology, University Hospital of Kinshasa, University of Kinshasa, Kinshasa, Democratic Republic of the Congo; Department of Epidemiology, Fielding School of Public Health, University of California, Los Angeles, California, USA

**Keywords:** Marburg virus disease, multiplex immunoassay, Democratic Republic of the Congo, serologic surveillance, filoviruses

## Abstract

**Background:**

To date, 18 Marburg virus disease (MVD) outbreaks have been reported, most recently in Tanzania and Ethiopia. From 1998 to 2000, a large MVD outbreak in Watsa/Durba region, Democratic Republic of the Congo (DRC), resulted in 154 cases and 128 deaths. Given the public health risks posed by MARV, the lack of approved vaccines and therapeutics, and unpredictability of future outbreaks, this study aimed to assess the anti-MARV seroprevalence in Watsa/Durba.

**Methods:**

Samples collected in May 2023 (n = 313) included known MVD survivors (n = 5), close contacts (n = 22), and other residents (n = 286). A multiplexed immunoassay was used to assess seroreactivity to MARV and other pan-filovirus targets.

**Results:**

Among the Watsa/Durba cohort, 4.5% and 1.6% were seroreactive to MARV glycoprotein (GP) and VP40, including only 1 known MVD survivor. No significant associations were found between MARV seroreactivity and risk factors: travel to Uganda, receiving injections, or employment as a gold miner. Of those 14 MARV GP-reactive individuals, 85.7% were cross-reactive to other filoviral targets.

**Conclusions:**

This detected seroprevalence among individuals with no known exposure history may indicate continued exposure to MARV or MARV-like antigens 25 years after the conclusion of the outbreak; cross-reactivity to pan-filovirus targets further suggest the potential for undetected cases.

## Teaser

Here, we detect anti-MARV antibodies among 313 residents of Watsa/Durba, DR Congo, 25 years after the last recorded outbreak. Low-level MARV and other pan-filovirus seroreactivity suggest ongoing exposure or underdetection of viral infections, underscoring the need for heightened surveillance.

*Orthomarburgvirus marburgense* (MARV) is the etiologic agent of Marburg virus disease (MVD), a rare, but potentially fatal, zoonotic hemorrhagic viral disease in both humans and nonhuman primates (NHPs) [1, 2]. Marburg virus disease belongs to the family *Filoviridae* and the genus *Orthomarburgvirus*; although closely related to ebolaviruses, it is classified in a distinct genus. The single species, *Orthomarburgvirus marburgense*, comprises 2 genetically distinct viruses—MARV and Ravn virus (RAVV) [3]. While most human cases have resulted from MARV infections, including those reported during the first outbreak in 1967, MARV and RAVV infections result in indistinguishable disease symptoms in both humans and NHPs [4]. Marburg virus disease was first reported following laboratory-associated MARV infections in laboratory workers in West Germany (Marburg and Frankfurt) and Serbia (formerly Yugoslavia; Belgrade) through contact with tissues from infected grivets (*Cercopithecus aethiops*). Since its first report, there have been 18 MVD outbreaks reported almost exclusively within Sub-Saharan Africa, with 6 reported between Uganda and the Democratic Republic of the Congo (DRC) [5]. Recent emergence events in Guinea, Ghana, Equatorial Guinea, Tanzania, and Rwanda indicate an expanding geographic range for MARV [1]. In December 2024, an MVD outbreak in Rwanda, close to a porous, shared border with DRC, resulted in 66 cases and 15 deaths [6]. Most recently, MVD was first reported in Ethiopia in November 2025, with 14 confirmed human cases [7].

*Orthomarburgvirus* zoonosis has been largely connected to human interactions with bat-infested areas, including caves and mines. While infectious MARV has been isolated from Egyptian rousette bats (*Rousettus aegyptiacus*), there is little information regarding natural routes of zoonotic transmission [8]. Subsequent genomic investigations of the 2024 MVD Rwanda outbreak identified limited genetic variation, indicative of a single spillover event. Further epidemiologic investigations of this outbreak linked the index case to a rousette bat-inhabited cave, providing further evidence of the link between the natural reservoir and continued human spillover events [9].

Clinical symptoms in humans generally occur between 2 and 21 days postinfection dominated by influenza-like symptoms, including fever, headaches, chills, myalgia, and general malaise that typically last 1–4 days. This is followed by a more intense phase from days 5 to 7 after onset of the disease, which can include maculopapular rash with progression to symptoms of hemorrhagic fever, multiorgan failure, and severe neurological symptoms. Disease survival is generally associated with less severe disease progression [10]. *Orthomarburgvirus marburgense* persistence within the male reproductive tract has been reported both in human patients and infected nonhuman primates; however, the risks for sexual transmission remain largely undetermined [11–13]. Additionally, pathologic abnormalities at the blood–testis barrier have been reported in MVD patients, including orchitis, contrasting with observations from reproductive tract persistence associated with *Orthoebolavirus zairense* (EBOV) [11]. *Orthomarburgvirus marburgense* transmission is driven primarily through contact with contaminated body fluids and/or tissues (including urine, saliva, feces, vomit, and semen), direct contact associated with traditional burial practices, or contact with contaminated objects, including bedding or medical instruments. There are currently no licensed vaccines or therapeutics available specific to MARV [2]. However, there are a number of vaccines that leverage recombinant filoviral glycoproteins in clinical trials that have shown promise [14, 15].

**Figure 1.**
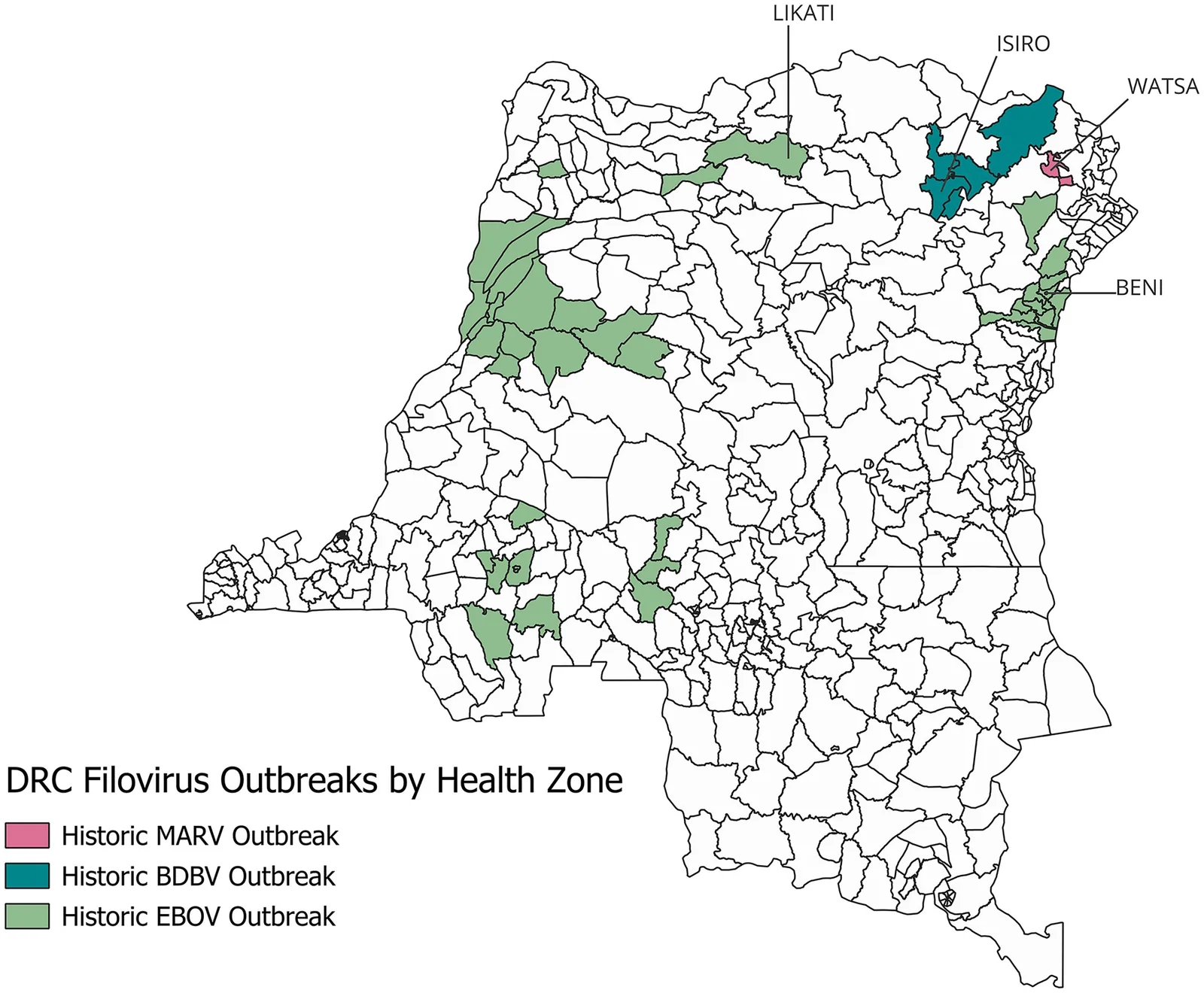
Geographic distribution of historic filovirus outbreaks in the Democratic Republic of the Congo. Cases are reported at the health zone (HZ) level (data sourced from internal situation reports [21–23],). Specific HZs are labeled to indicate nearby filovirus outbreak sites to Watsa HZ.

Case fatality rates (CFRs) for MVD have typically been high during large disease outbreaks, for example, 88% during the 2004–2005 Angola outbreak; a recent systematic review showed that the unadjusted, pooled total random effect case fatality ratio for MVD was 61.9% [4]. The most recent MVD outbreak in DRC occurred from 1998 to 2000 in the Watsa/Durba health zones and resulted in 154 confirmed cases and 128 deaths (83% CFR) [16]. Watsa is located in Haut-Uele, a northeastern province of DRC bordering South Sudan and Uganda. Due to frequent border crossings, proximity to wildlife, and high levels of displacement caused by ongoing geopolitical turmoil, there is a high risk of potential exposure to different filoviruses that have previously been detected in this region, including EBOV, *Orthoebolavirus sudanense* (SUDV) and *Orthoebolavirus bundibugyoense* (BDBV). Historically, many index cases of MVD outbreaks have reported exposures to caves in the region; cave concentration and established bat colonies in the region may be driving continued MARV exposure [17]. Further, this region is a hub for commercial and artisanal gold mining—an established risk factor of MVD [18]. Although only 1 MVD outbreak specifically has been confirmed in these health zones, historic EBOV and BDBV outbreaks in nearby Likati, Beni, and Isiro health zones may indicate potential exposure to other viruses of the same family in the region [19, 20] (Figure 1). While most recently a SUDV outbreak was reported in neighboring Uganda in January 2025, no SUDV outbreak has ever been reported in the DRC [24].

Surveillance studies have investigated seroprevalence rates and humoral immune response persistence to related pathogens in DRC, such as EBOV [25, 26], yet little is known about the seroprevalence to MARV, nor sustained antibody responses among known MVD survivors. Furthermore, without a registered anti-MARV vaccine or other specific medical countermeasures, understanding the natural history of infection, including potential correlates of protection and the durability of immune responses, is critical for MVD prevention and control. This study sought to assess population seroreactivity to 2 MARV antigens, as well as possible pan-filovirus reactivity among a cohort of known survivors and close contacts of the Watsa/Durba outbreak and healthcare workers in the region.

## METHODS

### Sample Collection

Samples for this cohort were collected as part of a larger study, which targeted regions in the DRC with historic EBOV and MARV outbreaks, as well as those considered at high risk for future outbreaks [27, 28]. As part of the Watsa-specific cohort collection in May 2023, inclusion criteria included consenting adults with a history of known MARV infection (as identified through Ministry of Health documentation, including survivors from the 1998–2000 outbreak), close contacts of known MARV survivors, and healthcare workers (HCWs) at the time of sampling in the region, who may or may not have participated in the MARV outbreak. All participants (adults, ≥18 years), were screened for fever by trained personnel and asked about symptoms associated with acute or recent illness, prior to each blood draw. Any individual presenting symptoms indicating illness at the time of sampling was excluded from the study. Following the enrollment of eligible participants, trained field staff administered a verbal questionnaire and conducted a brief physical examination, which was electronically recorded. All data were collected using electronic tablets and stored on a secured SurveyCTO server. Blood samples (approximately 10 mL) were collected and processed in field laboratories; serologic testing of stored sera was performed at the Institut National de Recherche Biomédicale (INRB), Kinshasa, DRC. During analysis, 2 observations were censored, as paired behavioral risk factor responses and demographic information were missing.

### Ethical Considerations

Ethical approval was obtained from the University of California, Los Angeles' Institutional Review Board (IRB#20-001091), and the Ethics Committee at the Kinshasa School of Public Health, University of Kinshasa, in the DRC (ESP/CE/142/2020).

### Sample Testing

Serum samples were tested in duplicate using a custom pan-filovirus multiplex immunoassay (MIA) to determine seroreactivity [29, 30]. The MIA pan-filovirus panel was composed of magnetic MagPlex microspheres (Luminex Corporation, Austin, TX) coupled with purified recombinant proteins, including EBOV glycoprotein (GP), MARV GP, BDBV GP, SUDV GP, EBOV nucleoprotein (NP), and both EBOV and MARV virus matrix protein 40 (VP40). The multiplex assay provides a higher throughput alternative to traditional enzyme-linked immunosorbent assay (ELISA) methods, where multiple filovirus targets can be evaluated concurrently [31]. Past investigations have validated the comparability of the EBOV GP target included this MIA with gold-standard immunofluorescence single plex techniques [30].

Bovine serum albumin (BSA) control beads were also included to determine nonspecific binding and background signal. All antigen median fluorescence intensity (MFI) responses were BSA adjusted by subtracting background reactivity values from raw MFI values. During BSA adjustment, MFI minimums were set to 0 to ensure no negative values were reported. Reactivity to unique antigens was determined by serum IgG binding to antigen-coupled microspheres. This binding was detected by a phycoerythrin-labeled goat antihuman IgG secondary antibody (Jackson ImmunoResearch Labs, West Grove, PA) and reported as registered fluorescence signal intensity as determined by the Luminex MAGPIX instrument. Additional protocol details have been previously described [14, 15, 29].

### Statistical Analysis and Reactivity Cutoffs

Statistical analyses were performed using SAS 9.4 (SAS Institute, Cary, NC) and R version 4.3.3 (“Angel Food Cake,” http://www.r-project.org). Associations between seroreactivity and potential risk factors were evaluated using Fisher's exact test, and data visualizations were produced using the “ggplot2” package. Seroreactivity cutoffs for each antigen were defined as the arithmetic mean of BSA-adjusted MFI values plus 3 standard deviations (SDs), based on data from 379 bio-banked Kinshasa participants presumed to be filovirus naive (Table 1). Participants in the Kinshasa control group were excluded if any MFI value exceeded 17 000 or if they were known filovirus survivors, close contacts of viral hemorrhagic fever (VHF) cases, or HCWs involved in previous filovirus outbreaks.

**Table 1.** MFI Cutoff Values as Calculated From a Population of MARV-Naive Kinshasa Inhabitants, Samples Collected in 2017

Target Antigen	Mean (MFI)	St. Dev (MFI)	Cutoff (MFI)
MARV GP	646	1394	4828
MARV VP40	1913	2430	9203
EBOV GP	168	807	2589
EBOV VP40	2744	2669	10 751
EBOV NP	613	1239	4330
SUDV GP	1872	2594	9654
BDBV GP	704	1243	4433

Reactivity cutoffs are calculated as the mean of BSA-adjusted MFI + 3 SDs of the naive Kinshasa based population (n = 379).

## RESULTS

### Binary Seroreactivity

The Watsa/Durba cohort (n = 313) included known MVD survivors of the 1998–2000 outbreak (n = 5), close contacts of those who were previously infected during a VFH outbreak (Ebola Virus Disease [EVD], or unspecified—n = 22), HCW (n = 269), and other individuals living in the health zone at the time of sampling in May 2023. In reporting binary seroreactivity to MARV GP and VP40 targets, 4.5% and 1.6% of participants were considered seroreactive, respectively (Table 2). Only 1 VP40-reactive individual was similarly MARV GP reactive—for all other VP40 reactive samples, no reciprocal MARV GP reactivity was detected. Five respondents had reported receiving an EBOV vaccination, of which 4 were seroreactive for EBOV GP, yet, when excluded from analysis, seroreactivity rates to all pan-filoviral antigens, except for EBOV GP and VP40, remained unchanged.

**Table 2.** Binary Seroreactivity to All Targets in a Multiplex Immunoassay Pan-filovirus Panel Among a Cohort of Samples Collected in the Watsa/Durba Region, 2023 (n = 313)

Target Antigen	Watsa/Durba Cohort (n = 313)		Respondents With No History of EBOV GP Vaccine (n = 308)	
	N	%	N	%
MARV GP	14	4.5	14	4.6
MARV VP40	5	1.6	5	1.6
EBOV GP	11	3.5	7	2.3
EBOV VP40	12	3.8	11	3.6
EBOV NP	3	1.0	3	1.0
SUDV GP	8	2.6	8	2.6
BDBV GP	45	14.4	45	14.6

**Figure 2.**
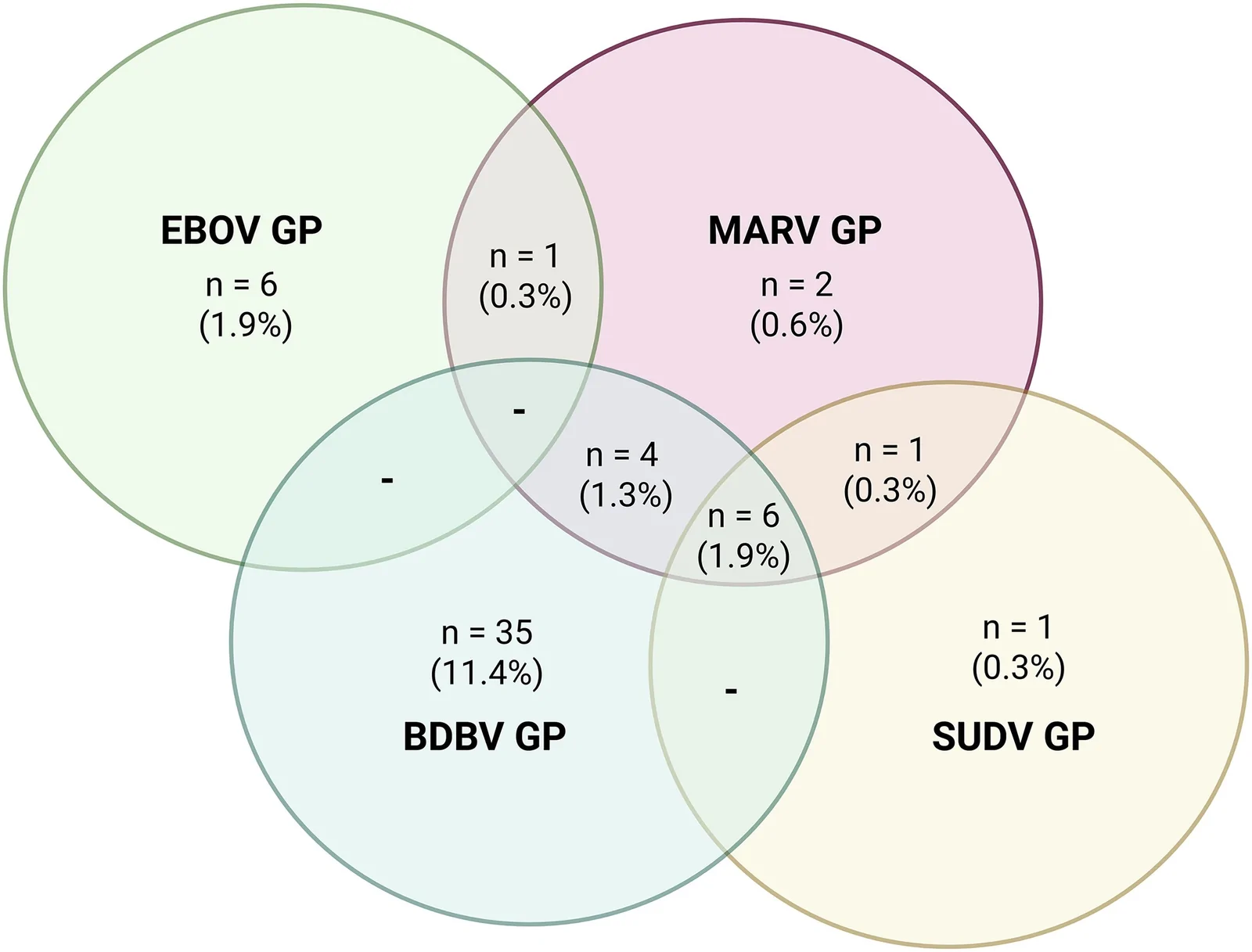
Pan-filovirus reactivity to MARV, EBOV, SUDV, and BDBV glycoprotein targets among the Watsa/Durba cohort collected 25 y after the last reported outbreak. Reported cross-reactivity excludes those individuals who reported receiving an EBOV vaccine. Percentages are reported as relative to the entire cohort population with no reported history of EBOV vaccination (n = 308).

Beyond MARV-specific reactivity, 2.3% of participants were seroreactive to EBOV GP and 14.6% to BDBV GP among those with no reported history of EBOV vaccination (n = 308). Notably, of the 14 individuals reactive to MARV GP, cross-reactivity to EBOV, SUDV, and BDBV targets was observed for 12 (85.7%) of these individuals (Figure 2).

Given the specific EBOV reactivity of those reporting a history of EBOV vaccination, these 5 individuals were removed from further analysis. Among this new cohort (n = 308), 7.1% of respondents reported close contact with a known EVD or other VHF patient, 6.8% reported recent travel to Uganda, and 4.2% were born after 1 October 2000, after the conclusion of the last reported MVD outbreak [16] (Table 3). Additionally, 2 individuals indicated that their primary occupation was gold mining—an occupation previously reported as a significant risk factor for MARV exposure [18]. Furthermore, 13 (4.2%) respondents reported receiving an injection during the course of the 1998–2000 outbreak—another known risk factor of MVD [18]. Frequency of reported zoonotic risk factors, such as consumption of wild game and contact with bats, was also recorded.

**Table 3.** Demographic Characteristics and Frequency of Selected Risk Factors of Watsa/Durba Cohort (n = 308)

	N	%
Age (mean, SD) (missing n = 5)	38.8	12.0
Age (binarized relative to 1998–2000 MVD outbreak)		
Born prior to outbreak	295	95.8
Born after outbreak	13	4.2
Sex		
Male	155	50.3
Female	153	49.7
Civil status		
Single	131	42.5
Married	118	38.3
Living with partner	33	10.7
Divorced or separated	12	3.9
Widowed	14	4.5
Primary occupation		
Healthcare worker (HCW)	269	87.3
Gold miner	2	0.6
Other	37	12.0
Were you considered a close contact of a confirmed EVD or viral hemorrhagic fever case?		
Yes	22	7.1
No	286	92.9
Have you ever been involved in an Ebola outbreak?		
Yes	67	21.8
No	241	78.2
Have you traveled abroad to Uganda in the last 6 m?		
Yes	21	6.8
No	287	93.2
During the most recent outbreak, did you receive an injection?		
Yes	13	4.2
No	295	95.8
In the last month, how frequently have you eaten bushmeat?		
Not in the past month	232	75.3
1–3 times per month	60	19.5
Once a week	10	3.2
More than once a week	6	1.9
In the last month, how frequently have you visited wooded/forested areas?		
Not in the past month	228	74.0
1–3 times per month	40	13.0
Once a week	11	3.6
More than once a week	29	9.4
In the last month, how frequently have you entered a cave or mine?		
Not in the past month	279	90.6
1–3 times per month	21	6.8
Once a week	5	1.6
More than once a week	3	1.0
In the last month, how frequently have you had contact with bats (not species specific)?		
Not in the past month	289	93.8
1–3 times per month	14	4.5
Once a week	4	1.3
More than once a week	1	0.3
In the last month, how frequently have you eaten nonhuman primate meat?		
Not in the past month	246	79.9
1–3 times per month	29	9.4
Once a week	9	2.9
More than once a week	24	7.8

### Seroreactivity Among Specific Subpopulations

**Figure 3.**
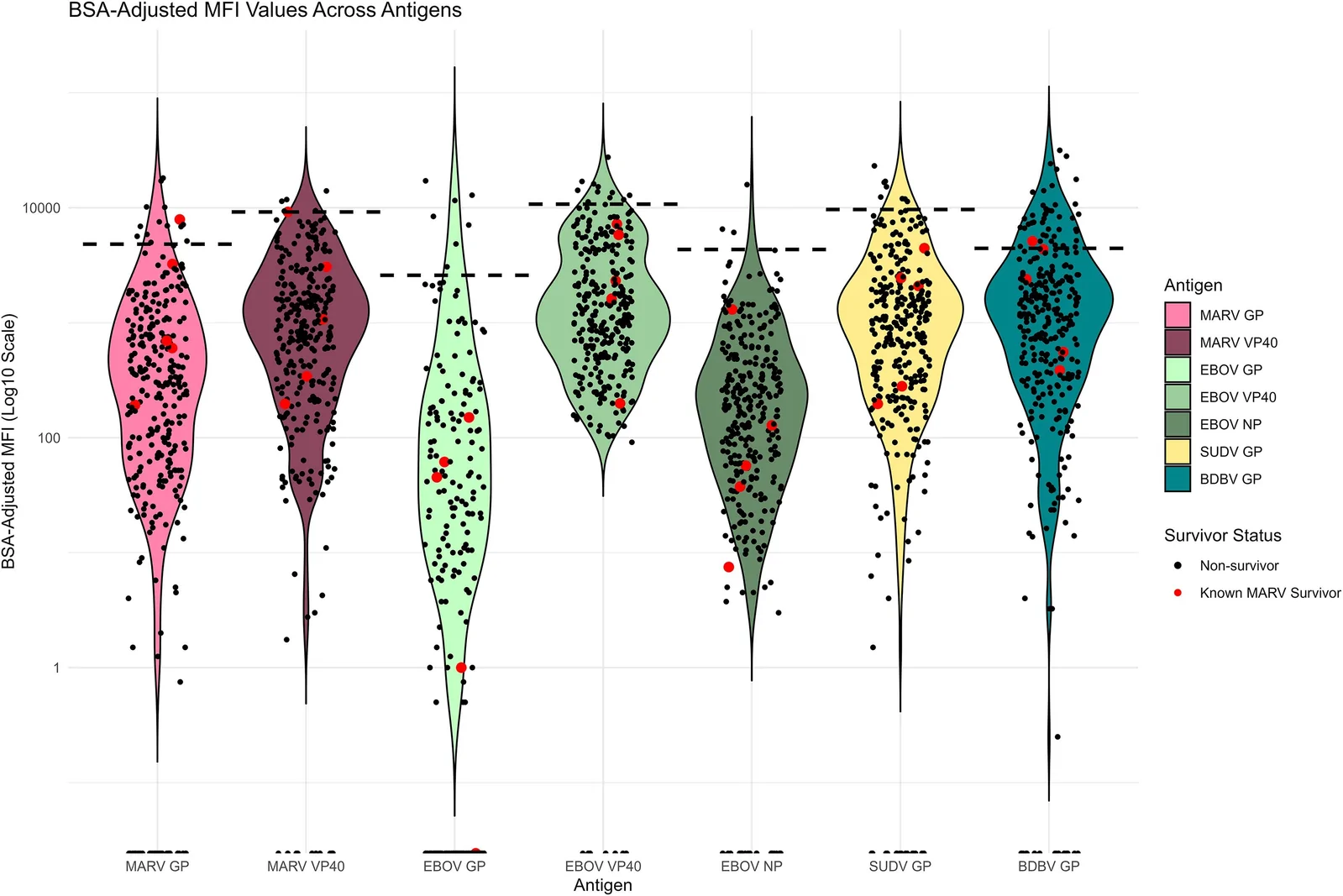
BSA-adjusted mean fluorescence intensity and established reactivity cutoffs to selected pan-filovirus antigens among the Watsa cohort (n = 308). Known MVD survivors are highlighted by the larger points, also in red; BSA-adjusted cutoffs are denoted by dashed lines.

When specifically assessing known MVD survivors, 1 of 5 was considered seroreactive to MARV GP, and no survivor was reactive to MARV VP40, based on the established cutoffs of this immunoassay. However, MFI can also be reported on a continuous scale, with relative increases in the fluorescence signal indicating changes in antibody reactivity. Of the 5 survivors, the MARV GP-reactive individual's MARV VP40 mean reactivity was 9200 MFI; while not considered seroreactive, this relative seroreactivity was not far from the set cutoff (Figure 3). Similarly, another known survivor had elevated antibody binding, but not considered seroreactive as detected by this immunoassay to MARV GP and VP40 at 3239 and 3068 MFI, respectively. In expanding to other panel targets, reactivity to BDBV GP was uniquely detected among 1 other known MARV survivor.

Of those respondents born after the Watsa/Durba MVD outbreak (post-October 2000), 1 individual was seroreactive to MARV GP; 2 of 13 (15.4%) were seroreactive to BDBV GP, one of whom was also cross reactive with SUDV GP. No reactivity to any EBOV, MARV, BDBV, or SUDV antigen target was reported among those participants who reported travel to Uganda (n = 21) in the past 6 months. Similarly, among those 2 respondents indicating that either their primary or secondary profession was gold mining, reactivity to BDBV GP was only observed in 1 individual.

### Known Marburg Virus Disease and Pan-filovirus Risk Factors

Using Fisher's exact test, associations between seroreactivity to MARV antigenic targets and known risk factors of both MVD and EVD were calculated. These risk factors were collapsed to binary yes/no for those behavioral questions with frequency responses (consumption of bushmeat, cave entry, etc.). While limited by the total number of seroreactive individuals, univariate analyses indicated an association between MARV GP seroreactivity among those participants who reported consuming bushmeat in the past month. Of note, univariate analyses also indicated associations between MARV VP40 reactivity and those participants who reported close contact with bats. Similarly, nonsignificant associations between seroreactivity and entering forested areas, consuming bushmeat, involvement in a previous EVD outbreak, and male respondents to all antigenic targets were indicated among the same cohort (Table 4). No associations were indicated between previous travel to Uganda and seroreactivity to any of the 5 filoviral targets. Many individuals had overlapping risk factors by nature of their occupation or zoonotic exposure; pairwise counts are presented in Supplementary Figure 1.

**Table 4.** Nonsignificant Associations Between Known Pan-filoviral Risk Factors and Seroreactivity to MARV GP, MARV VP40, EBOV GP, SUDV GP, and BDBV GP Targets

	N	%	MARV GP				MARV VP40				EBOV GP				BDBV GP				SUDV GP			
Characteristic/Risk Factor			OR	Lower CI	Upper CI	*P*-Value	OR	Lower CI	Upper CI	*P*-Value	OR	Lower CI	Upper CI	*P*-Value	OR	Lower CI	Upper CI	*P*-Value	OR	Lower CI	Upper CI	*P*-Value
Sex: male	155	50.3	1.33	0.39	4.78	.79	0.65	0.05	5.80	.68	0.74	0.11	4.43	.72	1.73	0.88	3.61	.11	3.03	0.53	31.16	.28
Occupation: healthcare worker	264	85.7	1.00	0.21	9.52	1.00	–	–	–	–	–	–	–	–	1.39	0.50	7.80	.65	1.17	0.14	54.00	1.00
Occupation: Gold miner	2	0.6	–	–	–	–	–	–	–	–	–	–	–	–	5.90	0.07	467.23	.27	–	–	–	–
Close contact of EVD or VHF case	22	7.1	1.00	0.02	7.31	1.00	–	–	–	–	–	–	–	–	1.33	0.31	4.31	.54	1.89	0.04	15.92	.45
Involved in the last EVD outbreak	67	21.8	0.98	0.17	3.86	1.00	5.56	0.62	67.97	.07	1.45	0.14	9.11	.65	1.19	0.51	2.61	.70	1.20	0.12	6.94	.69
Travel to Uganda in the last 6 m	21	6.8	–	–	–	–	–	–	–	–	–	–	–	–	–	–	–	–	–	–	–	–
Received injections at the time of last outbreak	13	4.2	–	–	–	–	–	–	–	–	–	–	–	–	1.07	0.11	5.14	1.00	–	–	–	–
*In the past month…*																						
Consumed bushmeat	76	24.7	0.50	0.05	2.31	.53	0.76	0.02	7.84	1.00	2.33	0.33	1.15	.37	0.73	0.29	1.65	.57	0.43	0.01	3.43	.68
Entered a forested/wooded area	80	26.0	0.21	0.00	1.44	.13	1.91	0.16	17.07	.61	1.14	0.11	7.16	1.00	1.04	0.46	2.22	1.00	0.40	0.01	3.20	.69
Entered a cave	29	9.4	–	–	–	–	2.45	0.05	25.87	.39	4.03	0.37	26.09	.13	0.41	0.05	1.72	.28	–	–	–	–
Close contact with bats (nonspecies specific)	19	6.2	–	–	–	–	3.93	0.08	42.53	.27	2.61	0.05	23.39	.36	0.31	0.01	2.07	.33	–	–	–	–
Consumed nonhuman primates	62	20.1	–	–	–	–	0.99	0.02	10.26	1.00	3.06	0.44	18.63	.15	0.45	0.13	1.22	.11	0.56	0.01	4.50	1.00

All *P*-values presented are those from Fisher's exact test. Those associations with no reported *P*-value were not calculated due to low cell counts.

## DISCUSSION

In summary, this serological survey in the Watsa/Durba region indicated that 4.5% of surveyed individuals were seroreactive to MARV GP nearly 25 years after the last recorded outbreak. Despite including known MVD survivors, only 1 survivor met the binary seroreactivity criteria for MARV GP, and none for MARV VP40, which may reflect limitations in the long-term durability of MARV antibodies. Officially, there were only 26 survivors of the 1998–2000 outbreak as recorded by official outbreak reports [5]—one limitation of this study was the low number of survivor participants. While only 19% of the known survivors were recruited, many were no longer in the Watsa/Durba region or were not able to be contacted by the study team at the time of sampling.

Beyond detection of antifilovirus immune responses, this study also assessed associations between detected seroreactivity and known risk factors of MVD specifically, as well as other reported risk factors of exposure to the filovirus family in general. While previous studies have documented gold mining in caves and receiving injections as significantly associated with MARV antibody detection [18], no significant associations between gold mining occupations, visiting caves, nor having received an injection in an outbreak setting, and MARV GP or MARV VP40 seroreactivity were observed in the study population. This could be due to relatively low population MARV seroreactivity and thus low cell counts to establish risk factor associations; of note, MARV antibodies may not be as durable as EBOV antibodies, which have been detected among survivors up to 40 years after an outbreak [26]. Among EVD survivors, we expect higher anti-EBOV GP titers as opposed to anti-MARV GP titers in MVD survivor cases, due to the production of secreted EBOV glycoproteins (sGPs) contributing to seroreactivity [32].

Additionally, when reporting risk behaviors, the questionnaire deployed was limited to actions taken within the last 6 months or in the context of an outbreak and might not adequately capture historical risks. While no significant associations were reported between cave entry or close contact with bats (a known reservoir) and MARV seroreactivity [33], those individuals with anti-MARV antibodies may have instead partaken in these activities outside of the “prior 6 months” time frame. Similarly, travel to Uganda and occupation variables (HCWs, gold mining) were only asked as current occupation, or within the last 6 months—this may not accurately represent historical exposure statuses or introduce recall bias among responses. Recall bias may also have played a role in determining close contacts of VHF cases, as well as risk behaviors at the time of the outbreak given that sampling and interviews were conducted over 20 years after the conclusion of the MVD outbreak.

Ultimately, the majority of seroreactive respondents did not report risk behaviors known to be associated with filovirus exposure and infection. As most of these individuals were not known survivors of MVD or any other tested filovirus, nor participated in activities that may increase the likelihood of exposure to known filoviruses, this may indicate exposure to a different MARV-like antigen, potential subclinical infection undetected by public health surveillance, or misdiagnosis. While no asymptomatic MVD infection has been documented in humans [2], this phenomenon has been documented in Egyptian rousette bats as a function of virus persistence and host immune tolerance [34]. Furthermore, known exposure history also has its limitations. For example, while a past household serosurvey of MVD close contacts in Watsa detected seroreactivity among 2 “unexposed” individuals, upon further investigation, both individuals had presented clinical symptoms of MVD—this just had not been documented [35].

Another consideration in the interpretation of these results is the variability of serologic results and the establishment of a seroreactivity cutoff when no international standard of antibody titers exists. For example, in using a BSA-adjusted mean plus 3 SD approach to set antigen-specific cutoffs from an assumed-naive Kinshasa cohort, only 1 MVD survivor was considered seroreactive. However, this does not indicate that those other survivors (n = 4) did not register any reactivity; relative MFI outputs may be considered lowly reactive, yet below the set threshold. Additionally, we saw little differences in our results or conclusions whether or not antigen MFIs were BSA adjusted or not. While a receiver operator curve (ROC) could be used to establish this threshold by designating known exposed and unexposed populations, this would be limited to confirmed MVD cases as known positives, and with such a limited population (n = 5), the set cutoff may not truly be reflective of anti-MARV titers as induced by past exposure to the virus.

As with any serologic approach, there are limitations to interpretations of detected seroreactivity. While the antigens used in this MIA have been used to develop promising recombinant vaccine candidates that have induced highly specific IgG titers in mice, guinea pigs, and NHPs [15], those detected seroreactive samples from Watsa should be assessed for specificity. Further investigations, such as neutralizing assays to evaluate the functionality of these detected anti-MARV antibodies, should be considered.

Of those individuals reactive to MARV GP, cross-reactivity to 1 or more different filovirus targets was observed for all but 2 participants (12 of 14). The pan-filovirus cross-reactivity observed in this cohort may have greater public health implications. While cross-reactivity and partial protection conferred by a recombinant vesicular stomatitis virus-vectored EBOV vaccine has been observed in Guinea pig models of SUDV [36], no cross-protection has been shown to date in NHP models or humans. Among BDBV disease survivors from nearby Uganda, monoclonal antibodies were cross-reactive to BDBV, SUDV, and EBOV targets with potent neutralizing activity among glycan-cap-specific antibodies; however, this cross-reactivity was not observed with MARV targets [37]. Notably, 87.3% of the study population were HCW; due to the geographic proximity of Watsa/Durba to other sites of BDBV (Isiro, Haut Uele) and EBOV (Beni, North Kivu) outbreaks, it is possible that more individuals were exposed through their occupation than the 67 who reported historical involvement in an EBOV outbreak. In this investigation, more than 85% of MARV GP-reactive respondents were cross-reactive to at least 1 additional filovirus GP target. Whether MARV—or potentially another virus altogether—exposure results in broader cross-reactive immune responses as compared to other Filoviridae family members should be investigated further. Additionally, the detection of EBOV GP immunoglobulin G seroreactivity is consistent with previous studies highlighting 18.7% anti-EBOV GP antibodies among the Efe pygmy population in the Watsa region [25]. Similarly, this study did not observe any significant association between detected seroprevalence and exposure to known risk factors.

Given the lack of a registered MARV vaccine or other medical countermeasures, and no recently reported outbreaks of MVD in DRC, the detected seroreactivity in individuals with no history of exposure to MARV may also indicate exposure to another pathogen. Additional investigations of MARV seroreactivity at a national scale may help to elucidate whether this observed seroreactivity is specific to Watsa/Durba or nationally representative in a country with a history of circulating filoviruses. Among the 5 survivors of this cohort, low MARV seroreactivity may also indicate waning antibodies, even after natural infection. This has major implications for evaluating population protection from future MARV or other filovirus exposure in a region with frequent outbreaks. Furthermore, the low-level reactivity to all other filovirus antigens included in this study highlights the critical need for, and importance of, strengthening surveillance in a region, such as the DRC, whether that be for known or unknown pathogens. With the possibility of undetected or subclinical cases of MVD—such as the 1 MARV GP seroreactive participant born after the conclusion of the last outbreak—these findings further underscore the value of national and regional public health systems to detect outbreaks and implement mitigation practices before larger, global spread.

## Supplementary Material

jiag155_Supplementary_Data

## Supplementary Data

Supplementary materials are available at *The Journal of Infectious Diseases* online (http://jid.oxfordjournals.org/). Supplementary materials consist of data provided by the author that are published to benefit the reader. The posted materials are not copyedited. The contents of all supplementary data are the sole responsibility of the authors. Questions or messages regarding errors should be addressed to the author.
