# Supplementary material for: Detection of Marburg Virus Antibodies 25 Years After Outbreak in Watsa, Democratic Republic of the Congo
Source: J Infect Dis. 2026 Mar 16;234(3):e724–33. doi: 10.1093/infdis/jiag155 (PMC13600397; doi:10.1093/infdis/jiag155)
Supplement: Supplementary file 1 [file jiag155_supplementary_data.zip › supp figure 1 legend.docx]

Supplementary Figure 1. Heatmap of pairwise overlap among selected risk factors. Percentages are reported as column percentages of each risk factor.
